# Prevalence of high cardiovascular risk by economic sector

**DOI:** 10.1007/s00420-019-01458-9

**Published:** 2019-07-15

**Authors:** Godelieve J. M. Vandersmissen, M. Schouteden, C. Verbeek, S. Bulterys, L. Godderis

**Affiliations:** 1 Group IDEWE, External Service for Prevention and Protection at Work, Interleuvenlaan 58, 3001 Heverlee, Belgium; 2 Katholieke Universiteit Leuven, Occupational, Environmental and Insurance Medicine, Kapucijnenvoer 35/5, 3000 Leuven, Belgium

**Keywords:** Cardiovascular disease, Cardiovascular risk, Economic sectors, Health promotion

## Abstract

**Purpose:**

The aim of this study was to assess the prevalence of high cardiovascular risk and the trend of cardiovascular risk factors in a large sample of Belgian workers.

**Methods:**

A cross-sectional study was performed on the data of workers under medical surveillance by the non-profit occupational health service IDEWE in 2018. The prevalence of poor health for smoking, physical activity, body mass index (BMI), and blood pressure according to the American Heart Association (AHA) definition was investigated. The presence of three or more poor cardiovascular health metrics was considered high cardiovascular risk. A log-binomial regression model was used to compare the prevalence of high cardiovascular risk between economic sectors taking into account age and gender and to calculate predicted probabilities of high cardiovascular risk.

**Results:**

Data about 212,792 workers were available. In 2018, overall, 7% of workers had high cardiovascular risk. Transport and construction had the highest prevalence of high cardiovascular risk, 14% and 12%, respectively. The lowest prevalence, 3%, was observed in education. Differences between sectors remained statistically significant after adjustment for age and gender. In men, workers in transport and storage and in construction had the highest predicted probability of high cardiovascular risk that increased with age. In women, highest predicted probability was observed in transport and storage.

**Conclusions:**

When implementing health promotion initiatives, priority should be given to sectors and professions where risk factors are most prevalent or are increasing rapidly. Measures should be tailored to the special needs of the occupational groups at high risk.

**Electronic supplementary material:**

The online version of this article (10.1007/s00420-019-01458-9) contains supplementary material, which is available to authorized users.

## Introduction

Although cardiovascular disease (CVD) mortality is decreasing in most European countries, CVD remains the leading cause of mortality and a major cause of morbidity on this continent. Each year, CVD causes 3.9 million deaths in Europe and 1.8 in the European Union (EU) (Wilkins et al. 2017). In 2015, CVD was responsible for the loss of 26 million Disability-Adjusted Life Year (DALYs) in the EU. DALYs are defined as years of healthy life lost due to disease and are calculated as the sum of years lost due to premature death and years lived with disability (Wilkins et al. 2017). Overall, CVD is estimated to cost the EU economy €210 billion a year. Around 26% (€54 billion) of this cost is due to productivity losses (Wilkins et al. 2017).

Major risk factors for CVD are smoking, high blood pressure, high cholesterol, obesity, diabetes, unhealthy diet, sedentary behaviour, and insufficient physical activity. Other risk factors are psychosocial and socio-economic factors like depression or low socio-economic status (Piepoli et al. 2016). There is also evidence that work-related factors contribute to CVD, directly or indirectly by influencing the above-mentioned major risk factors. These work-related factors may be physical, chemical, or psychosocial. Shiftwork, passive smoking, and job strain, among others, are well documented in this respect (Kristensen 1989a, b; Nyberg 2013; Olsen and Kristensen 1991). Therefore, it is not surprising that the prevalence of CVD and cardiovascular risk factors differs between professions and economic sectors (Kelsall et al. 2018; MacDonald et al. 2017; Shockey et al. 2016; Zimmermann Verdejo et al. 2010). An economic sector includes a particular range of occupations, which may involve a higher risk for CVD or for some cardiovascular risk factors because of the associated working conditions or the socio-economic background of the workers which they attract.

European cardiovascular health statistics show that there is a downward trend in risk factors such as smoking, high cholesterol, and hypertension, while other cardiovascular risk factors, particularly overweight and diabetes, have increased considerably (Timmis et al. 2018; Wilkins et al. 2017). These statistics concern general populations, but no information is available on the evolution of these cardiovascular risk factors in the working population.

Nevertheless, insight into the evolution of cardiovascular risk factors and the burden of cardiovascular risk per economic sector is of particular importance to focus work place health initiatives on the groups that may benefit most. Moreover, knowing the risk factors involved and their work-relatedness helps to direct the health interventions needed for a particular occupational group. Tailoring health promotion programmes to address specific needs is one of the key elements of successful workplace health interventions (Steel et al. 2018).

In 2010, the American Heart Association (AHA) defined a concept of ideal cardiovascular health to monitor cardiovascular health in the US population. For this purpose, they defined poor, intermediate, and ideal health for four health behaviours [diet, physical activity, smoking, and body mass index (BMI)] and three health factors (blood pressure, total cholesterol, and blood glucose) (Lloyd-Jones et al. 2010). This concept has been used in a limited number of studies involving US working populations. These studies confirmed the relationship between occupation and cardiovascular health (MacDonald et al. 2017; Shockey et al. 2016). However, there are limited data on CVD risk factors in European working populations. A Spanish (Madrid region) study, which used the other methods of estimating cardiovascular risk, found a significant relationship between cardiovascular risk and occupation (Zimmermann Verdejo et al. 2010).

The aim of this study was to assess the prevalence of high cardiovascular risk in a large sample of Belgian workers as well as its relationship to demographic characteristics and economic sectors. In addition, the trend of cardiovascular risk factors during the last two decades was investigated.

## Methods

A cross-sectional study was performed on the medical data of workers under medical surveillance by IDEWE, a non-for profit external service for prevention and protection at work, in 2018. IDEWE is the largest Belgian occupational health service, covering more than 20% of Belgian workers. About 30% of workers under the medical surveillance of IDEWE are from large enterprises of 500 employees or more, while 25% are from small businesses of less than 50 employees.

In Belgium, periodic health surveillance is mandatory for workers exposed to occupational hazards, and includes a periodic, mostly yearly, examination. Belgian employers who do not have an internal occupational health service can choose between a dozen external occupational health services. More details on the Belgian occupational health system are described by Godderis et al. (2014). In 2018, 222 occupational health nurses and 179 occupational physicians from IDEWE performed medical examinations and recorded data in the workers’ electronic medical files. The data stored in the electronic medical files were loaded into a data warehouse described by Godderis et al., according to Belgian and international privacy and ethical legislation, allowing post hoc analysis of anonymised data (Godderis et al. 2015).

Only employees that had a regular health check during a periodic examination or at recruitment were included in the investigation. Data collected during these examinations included lifestyle habits such as smoking and physical activity, and biometric measures such as height, weight, BMI, and blood pressure. For blood pressure measurement, automated as well as aneroid sphygmomanometers were used. All measuring equipment was calibrated according to ISO 9001 certification standards. Demographic characteristics (date of birth and gender) were encoded per worker. The statistical Classification of Economic Activities in the European Community or Nomenclature statistique des Activités économiques dans la Communauté Européenne (NACE) was used to characterise the economic sector in which the worker was employed. For the analysis, the NACE codes were regrouped in main categories as described by Godderis et al. (2015).

In 2018, data concerning 214,280 employees were available. After data cleaning and exclusion of cases for whom data on age, gender, economic sector, smoking status, physical activity, BMI, or blood pressure were missing, data concerning 212,792 workers remained for analysis. We used the data in our database to replicate as closely as possible the AHA definitions of poor health for smoking, physical activity, BMI, and blood pressure (Lloyd-Jones et al. 2010). The three other AHA health metrics—dietary habits, blood glucose, and total cholesterol—were not routinely assessed and measured, and were not included in this analysis. An overview of the definitions of the four health metrics used in this study is shown in Table 1.

**Table 1:** Definition of poor and non-poor health for smoking, physical activity, body mass index, and blood pressure

Metric	Measurement for current study	Categories	
		Poor health	Non-poor health
Smoking	Interview by nurse or physician	Current smoker	Never or former smoker
Physical activity	Interview by nurse or physician about leisure time physical activity	None	Any level
Body mass index	Measurement of length and weight (without shoes) by nurse	≥ 30 kg/m^2^	< 30 kg/m^2^
Blood pressure	Measurement by nurse or physician	SBP ≥ 140 mmHg or DBP ≥ 90 mmHg with or without antihypertensive treatment	SBP < 140 mmHg or DBP < 90 mmHg with or without antihypertensive treatment

*SBP* systolic blood pressure, *DBP* diastolic blood pressure

The more risk factors which a person has and the poorer each risk factor, the higher the chance that a person develops CVD (Piepoli et al. 2016). A “high cardiovascular risk” group was defined as those workers meeting three or four “poor” health metrics. A trend analysis of the same cardiovascular health metrics was performed. For this purpose, cross-sectional data, gathered during the medical examinations of workers under the medical surveillance of IDEWE since 1993, were used. The method of data selection was the same as described for the data from 2018. The number of workers in the study populations from 1993 to 2018 is shown in supplementary table 1. The prevalence of smoking and obesity from 1993 until 2018 was plotted over time. Blood pressure and physical activity data were only available from 2011 onwards. Therefore, for hypertension and physical inactivity, the plots started from 2011.

Statistical analyses were conducted with SPSS 19.0 for Mac and R (version 3.6.0) including packages Epi (version 2.37) and Foreign (version 0.8-71). Chi-square with Bonferroni correction was used to test for statistically significant differences between gender and age categories and between economic sectors. The prevalence of high cardiovascular risk was analysed by economic sector, adjusted for age and gender using a log-binomial regression model as described by Espelt et al. (2017). For this purpose, cardiovascular risk was dichotomised as follows: 1 or “high risk” if 3 or 4 poor health metrics, 0 or “no high risk” if only 2, 1, or no poor health metrics. The categorical variables, gender and sector, were dummy coded with female and services as reference group. A log-binomial regression model was built with the three main variables and all the possible two-way interactions. All interactions turned out to be statistically significant. Therefore, these were kept in the definite model beside the three main variables (supplementary table 2). The predicted probabilities of high cardiovascular risk by age and by sector obtained by this model were visualised in scatterplots for men and women.

## Results

### Demographic characteristics

The demographic characteristics of the study population and per sector are described in Table 2. Fifty-five percent were male; mean age was 40.8 years with a minimum of 15 years and a maximum of 69. Gender and age distribution varied among sectors. Almost all workers in construction and in transport and storage were male. Mean age was highest in transport and storage (44.2 years) and lowest in education (35.2 years).

**Table 2:** Gender and age distribution by sector

Economic sector	*n*	Male (%)	Female (%)	Mean age (SD)
Education	12,911	33.4	66.6	35.2 (14.1)
Health care	64,986	19.0	81.0	41.3 (12.1)
Government	28,796	58.8	41.2	42.6 (11.6)
Accommodation and food service	2873	47.1	52.9	39.9 (12.4)
Distributive trade	22,403	70.0	30.0	39.0 (11.9)
Manufacturing	32,228	85.4	14.6	42.1 (11.2)
Services	18,096	68.3	31.7	38.4 (12.2)
Construction	9952	98.7	1.3	39.2 (11.6)
Transport and storage	11,900	91.0	9.0	44.2 (11.1)
Other	8647	69.7	30.3	42.0 (11.9)
Total	212,792	55.1	44.9	40.8 (12.1)

### Cardiovascular health metrics

In Table 3, the prevalence of poor health for the four metrics in the study population is shown as well as their prevalence by gender, age, and economic sector. Physical activity was the health metric that was most frequently poor (33%), followed by smoking (26%).

**Table 3:** Prevalence of poor health for each metric in the study population and by gender and age categories and by economic sectors

	Smoking		Physical activity		BMI		Blood pressure	
	Poor health		Poor health		Poor health		Poor health	
	%	p*	%	p*	%	p*	%	p*
Study population	26.1		33.3		19.1		19.2	
Gender		< 0.001		< 0.001		< 0.001		< 0.001
Male	31.8		34.8		19.8		23.7	
Female	19.1		31.4		18.3		13.6	
Age categories		< 0.001		< 0.001		< 0.001		< 0.001
15–24 years	27.4		34.2		11.5		9.3	
25–34 years	28.8		32.7		14.4		11.0	
35–44 years	28.0		35.0		19.6		16.4	
45–54 years	23.9		34.0		23.9		25.5	
55–69 years	21.9		29.8		22.5		31.4	
Economic sectors		< 0.001		< 0.001		< 0.001		< 0.001
Education	14.9		26.4		15.0		14.4	
Health care	19.1		27.9		18.4		15.7	
Government	22.6		25.5		19.8		17.3	
Accommodation and food services	36.8		42.5		17.7		18.2	
Distributive trade	31.4		42.1		17.9		21.1	
Manufacturing	30.8		35.7		18.9		21.4	
Services	33.3		38.9		17.6		19.2	
Construction	40.7		46.1		19.8		25.4	
Transport and storage	35.3		41.8		30.6		30.7	
Other	27.2		35.8		18.9		22.1	

*p resulting from Chi-square test with Bonferroni correction

For all metrics, poor health was more frequently present in male than in female. The prevalence of poor health for blood pressure increased with age. The prevalence of poor health for BMI increased with age as well, but was lower in workers of 55 years or older than in the age category 45–54 years. Poor health for smoking and physical activity was most frequent in younger age categories, 25–34 years and 35–44 years, respectively.

Comparing economic sectors, poor health for BMI and blood pressure were most present in the transport and storage sector. Almost one-third of workers in transport and storage had a BMI of 30 or over and had hypertension. Construction was the leader in poor health for smoking and physical activity and was second in poor health for blood pressure. Education was the sector with lowest prevalence of poor health for smoking, BMI, and blood pressure, and also had second lowest prevalence of poor health for physical activity.

### High cardiovascular risk

**Fig. 1:**
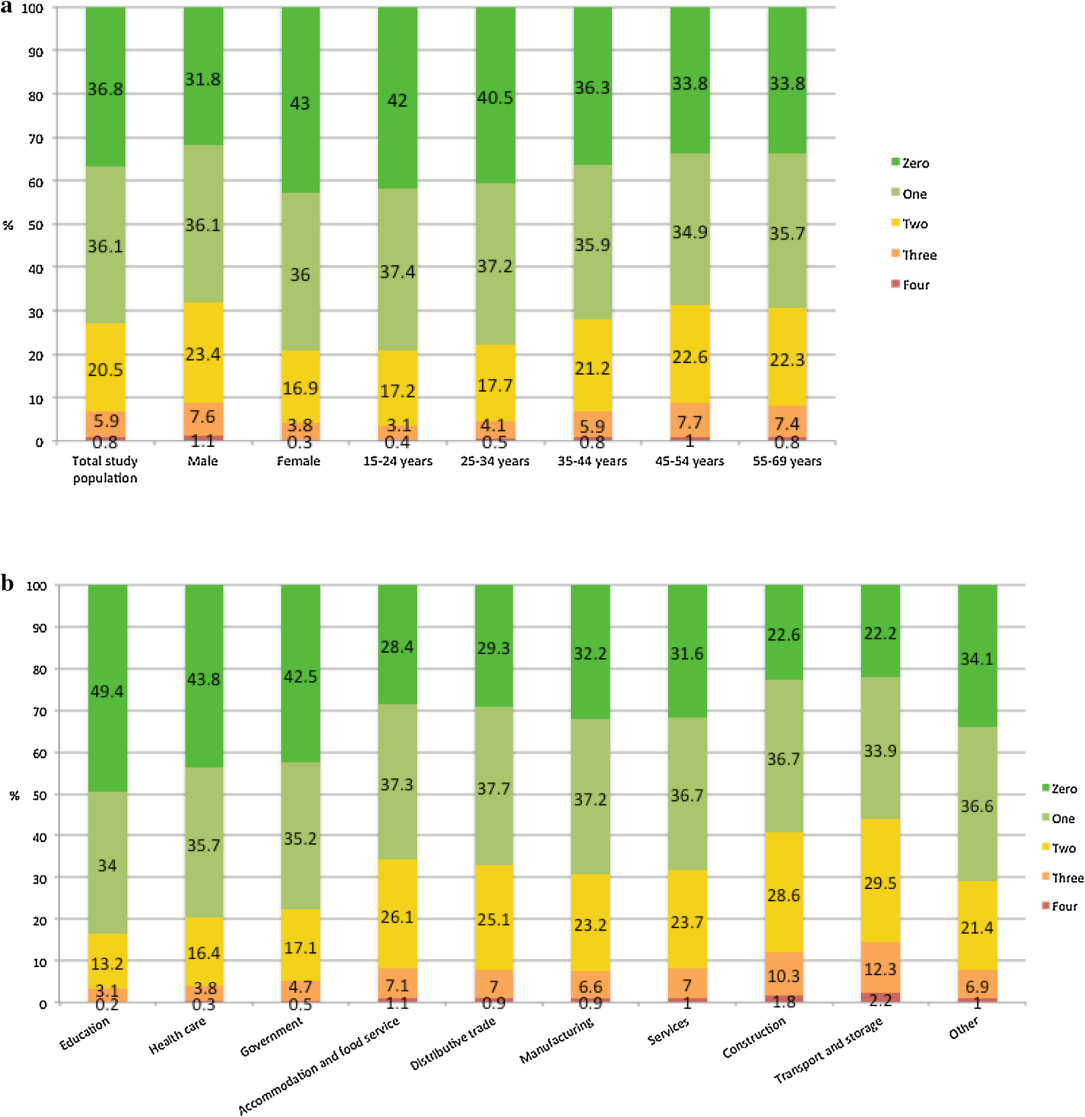
**a** Distribution of workers by number of poor health metrics in the total study population and by gender and age category. Chi-square with Bonferroni correction, testing the relationship between number of poor health metrics and gender: *p* < 0.001. Chi-square with Bonferroni correction, testing the relationship between number of poor health metrics and age category: *p* < 0.001. **b** Distribution of workers by number of poor health metrics by economic sector. Chi-square with Bonferroni correction, testing the relationship between number of poor health metrics and sector: *p* < 0.001

In Fig. 1a, b, the distribution of the workers according to the number of poor health metrics is shown for the study population and by gender, age category, and economic sector. Seven percent of workers had three or four poor cardiovascular health metrics, indicative for high cardiovascular risk. As to be expected, high cardiovascular risk was statistically significantly associated with gender and age. The proportion of workers with high cardiovascular risk was 9% in male and 4% in female. It increased from 3% in workers 15–24 years to 9% in those 45–54 years and fell back to 8% in the oldest age group. The latter observation is in accordance with the lower prevalence of poor health for smoking, physical activity, and BMI in the oldest age group in comparison to those of 45–54 years old. In addition, there was a statistically significant difference in the prevalence of high cardiovascular risk between economic sectors. Highest cardiovascular risk was observed in transport and storage, 14%, followed by construction with 12%. Lowest prevalence was observed in education, only 3%.

Because gender and age distribution differed significantly between sectors, a log-binomial regression model was used to adjust the relationship between high cardiovascular risk and economic sector for these variables. All significant interactions were included in the final model. This multiple log-binomial regression analysis (supplementary table 2) revealed that the differences in high cardiovascular risk between sectors remained statistically significant after adjustment for gender and age.

**Fig. 2:**
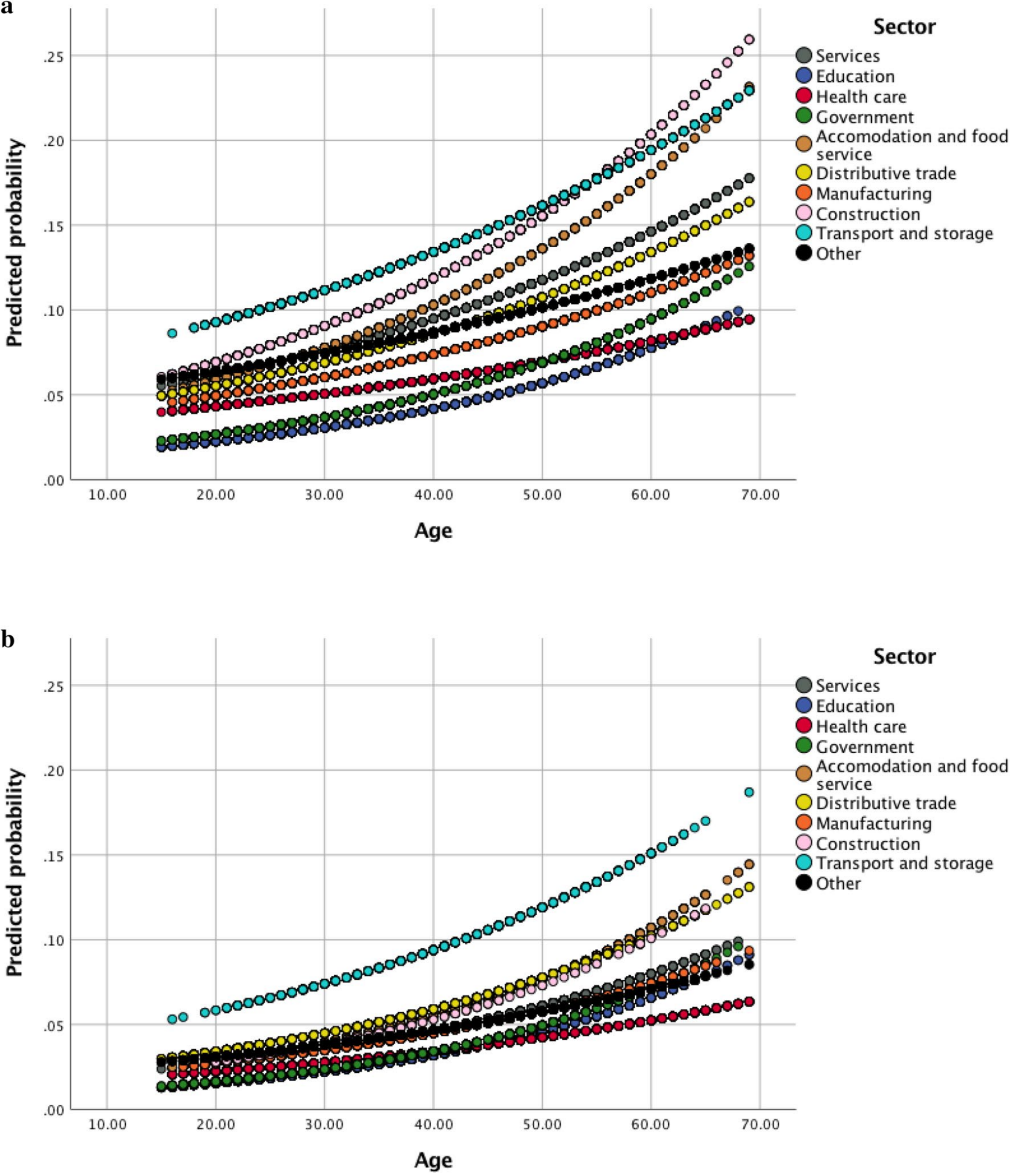
**a** Predicted probability of high cardiovascular risk (three or four poor cardiovascular health metrics) by age and by sector for men. **b** Predicted probability of high cardiovascular risk (three or four poor cardiovascular health metrics) by age and by sector for women

From the graphs (Fig. 2a, b) of the predicted probabilities of high cardiovascular risk by age and sector, which were obtained by the regression model, we can conclude that, in men, workers in the transport and storage and in the construction sectors had the highest probability of high cardiovascular risk that increased with age. In women also, workers in transport and storage had the highest probability of high cardiovascular risk. Lowest probabilities of high cardiovascular risk by age in both sexes were observed in the education, health care, and government sectors.

### Trend

**Fig. 3:**
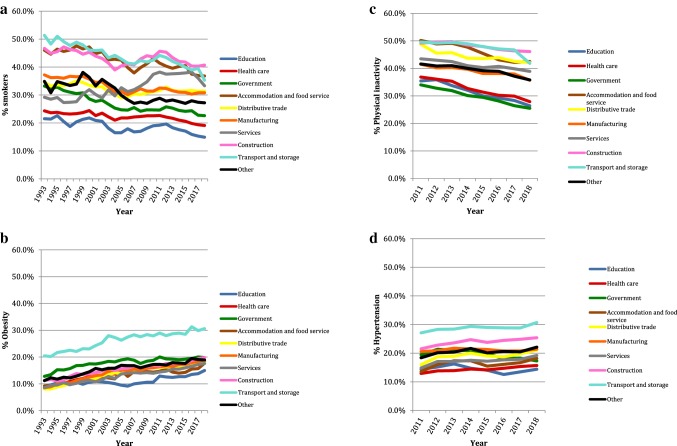
**a** Trend of the prevalence of smoking by sector in time. **b** Trend of the prevalence of obesity by sector in time. **c** Trend of the prevalence of physical inactivity by sector in time. **d** Trend of the prevalence of hypertension by sector in time

The trend of poor health for the four cardiovascular health metrics involved is shown in Fig. 3a–d. Physical inactivity was decreasing in all and smoking in almost all economic sectors. Services were the only sector in which the prevalence of smoking was higher in 2018 than in 1993. Hypertension was increasing in most economic sectors from 2011 onwards. Only in the government sector was the prevalence of hypertension lower in 2018 than in 2011. Obesity, however, was increasing in all sectors from 1993 onwards.

## Discussion

We found a considerable number of Belgian workers with high risk for CVD. Overall 7% had three or more poor cardiovascular health metrics according to the AHA’s definitions. The prevalence of high cardiovascular risk differed significantly according to sector, even after adjustment for gender and age.

There are some limitations to this study. First, this study has a cross-sectional design. Therefore, it is not possible to demonstrate a causal relationship. Second, 401 health professionals were involved in data collection. Although there were clear guidelines for the interviews about and registration of physical activity and smoking behaviour, and calibrated equipment was used to measure length, bodyweight, and blood pressure, there is always some chance of inter-observer variation. However, as observers were randomly assigned to different sectors, we expect that observer variation did not influence the sector analysis. Third, the rounding-up of blood pressure measurements to tens could have resulted in an overestimation of poor health status for blood pressure. The error that could arise from this inaccuracy in registration is less a problem in comparing groups, as we expect the fault is of the same size for all groups. Fourth, the vast majority of workers in this study were exposed to occupational hazards. Therefore, prevalences of poor health metrics in this sample might be higher as compared to the general working population. Finally, healthy worker effect probably influenced the results. The decrease of high cardiovascular risk in the oldest age group might be due to this effect. The strengths of this study include the large sample size and the use of data that are not self-reported but obtained by interview and measured by trained health professionals.

Highest prevalence of three or more poor cardiovascular health metrics was observed in the transport and storage and in the construction sectors, 14% and 12% respectively. These findings are consistent with those of a large-scale Australian study that evaluated the Australian Absolute Cardiovascular Risk Score according to industry. They found transport and construction to be among those industries with highest prevalence of high cardiovascular disease risk (Kelsall et al. 2018).

Forty-two percent of workers in transport and storage in our study did not take part in any leisure time physical activity, 31% were obese and 31% had hypertension. Similar to these findings, an analysis of CDC of data from the 2013 Behavioural Risk Factor Surveillance System (BRFSS) by occupation that considered the seven AHA cardiovascular health metrics obtained by telephone survey demonstrated that transportation and material moving workers had the highest prevalence of “not ideal” scores for physical activity, blood pressure, and body mass index. In this CDC study, responses for each of the seven cardiovascular health metrics were scored “not ideal” or “ideal”. In addition, they found workers in transportation and material moving had the second highest prevalence of 3, 4, 5, 6, or 7 “not ideal” cardiovascular health metrics (Shockey et al. 2016). A Spanish study using data from the Surveillance System for Non-transmissible Diseases Risk Factors (SIV-FRENT) for the Madrid region found that drivers accumulated more than two cardiovascular risk factors more often than other occupations (Zimmermann Verdejo et al. 2010). An analysis of data from our data warehouse on health complaints, medication, and sickness absence by economic sector between 2010 and 2014 revealed that transport and storage were the top two for cardiovascular health problems and cardiovascular medication (Godderis et al. 2015). One reason for these findings could be the sedentary aspect of driving that may attract an older and less active population. On the other hand, the daily life of many drivers involves long hours of sitting and consumption of unhealthy food inducing insufficient physical activity and obesity.

In our study, the prevalence of high cardiovascular risk was also high in the construction sector, especially in males. Construction was the leader in poor health for smoking: 41% of workers were smokers. In addition, a large number, 25%, had hypertension. Hypertension is more prevalent in males than in females and in older persons. However, the high prevalence of hypertension which we found in the construction sector cannot be explained by these factors. Hypertension was more prevalent in the construction sector than in males overall in this study despite a lower mean age of workers in construction than overall. High prevalence of hypertension and other cardiovascular risk factors was also observed in construction workers in Hong Kong (Yi and Chan 2016). One explanation may be that construction workers have a higher level of occupational physical activity compared to leisure time physical activity (Gram et al. 2016). Moreover, their occupational activity consists largely of manual material handling tasks (Gram et al. 2016; Hartmann and Fleischer 2005; Tak et al. 2011). Static occupational physical activities such as lifting heavy loads are known to cause sustained elevated blood pressure (Clays et al. 2012; Holtermann et al. 2018).

It would be interesting for future research to investigate cardiovascular risk in specific professional groups (for instance, professional drivers and construction workers), more specifically the occupational, lifestyle, and socio-economic factors that are involved and how they interact. As described in European CVD statistics, our study showed a downward trend of smoking, except in the services sector where smoking increased from 1993 onwards (Timmis et al. 2018; Wilkins et al. 2017). We also observed a worrying permanent growth in obesity in workers over the last two decades. This corresponds to the increase in mean BMI and in the prevalence of diabetes observed in Europe in recent decades (Timmis et al. 2018; Wilkins et al. 2017). The decrease in physical inactivity in all sectors seems contrary to the increase in obesity. A possible explanation might be that a decrease in inactivity is not equal to an increase in physical activity that is large enough to have health-enhancing effects. Contrary to European statistics, we observed an increase in the prevalence of hypertension, except for the government sector where the prevalence of hypertension was lower compared to 2011 (Timmis et al. 2018; Wilkins et al. 2017). The increase in the prevalence of hypertension in most sectors is consistent with the rise in obesity. As far as the government sector is concerned, the prevalence of obesity did not increase but stabilised from 2011 onwards. A factor that might play a role in a possible discrepancy between the obesity trend and the hypertension trend is the adherence to antihypertensive medication, which may be better in certain populations leading to better control of blood pressure.

## Conclusion

In Belgium, there is a substantial difference in the prevalence of high cardiovascular risk, defined as the presence of three or more poor cardiovascular health metrics, between economic sectors. Highest prevalence was observed in the transport and storage and the construction sectors. From this, we can assume that sector-related working conditions influence major cardiovascular risk factors. These working conditions might be physical, for example high static physical workload in construction workers, or organisational, for example not easily accessible healthy food for professional drivers. Therefore, more attention should be paid to the impact of work-related factors on cardiovascular health and to the actions that have to be taken to address them. This could be, for example, sufficient recovery time for workers with high static workload or healthy lifestyle interventions tailored to the specific situation of professional drivers.

Addressing major cardiovascular risk factors such as the further reduction of smoking and putting a stop to the growing epidemic of obesity is obviously a challenge for workplace health promotion. Priority should be given to sectors and professions where these risk factors are most prevalent or are increasing rapidly. Moreover, it is necessary to ensure that the health-promoting measures are tailored to the specific needs of the occupational groups at high risk.

## Electronic supplementary material

Below is the link to the electronic supplementary material.

Supplementary file1 (DOCX 49 kb)
