# Supplementary material for: Prevalence of high cardiovascular risk by economic sector
Source: Int Arch Occup Environ Health. 2019 Jul 15;93(1):133–42. doi: 10.1007/s00420-019-01458-9 (PMC6989576; doi:10.1007/s00420-019-01458-9)
Supplement: Supplementary file 1 — Supplementary file1 (DOCX 49 kb) [file 420_2019_1458_MOESM1_ESM.docx]

**Supplemetary table 1**

Number of workers in the study population from 1993 to 2018

| Year | n |
| --- | --- |
| 1993 | 79 940 |
| 1994 | 75 424 |
| 1995 | 83 043 |
| 1996 | 86 982 |
| 1997 | 94 955 |
| 1998 | 105 284 |
| 1999 | 110 922 |
| 2000 | 114 110 |
| 2001 | 112 229 |
| 2002 | 120 850 |
| 2003 | 118 632 |
| 2004 | 121 360 |
| 2005 | 108 963 |
| 2006 | 124 575 |
| 2007 | 146 849 |
| 2008 | 172 340 |
| 2009 | 176 760 |
| 2010 | 193 798 |
| 2011 | 217 036 |
| 2012 | 221 460 |
| 2013 | 218 280 |
| 2014 | 223 877 |
| 2015 | 226 091 |
| 2016 | 217 303 |
| 2017 | 209 718 |
| 2018 | 212 792 |

**Supplementary table 2**

Log-binomial regression model with high cardiovascular risk (3 or 4 poor cardiovascular health metrics) as outcome and age, gender, sector, gender*age, sector*age and gender*sector as dependent variables

|  | **B** | **SE** | **Wald** | **p** | **Exp(B)** | **95% CI for Exp (B)** |
| --- | --- | --- | --- | --- | --- | --- |
|  |  |  |  |  |  |  |
| Age | 0.027 | 0.003 | 10.527 | <0.001 | 1.027 | 1.022 - 1.032 |
| Gender (reference = female) | 0.922 | 0.104 | 8.907 | <0.001 | 2.513 | 2.052 - 3.079 |
| Sector (reference = services) |  |  |  |  |  |  |
| Education | -0.756 | 0.191 | -3.963 | <0.001 | 0.470 | 0.323 - 0.682 |
| Health care | -0.084 | 0.136 | -0.616 | 0.538 | 0.920 | 0.704 - 1.201 |
| Government | -0.709 | 0.160 | -4.445 | <0.001 | 0.492 | 0.360 - 0.673 |
| Accommodation and food service | -0.087 | 0.281 | -0.308 | 0.758 | 0.917 | 0.529 - 1.591 |
| Distributive trade | 0.214 | 0.143 | 1.496 | 0.135 | 1.239 | 0.936 - 1.639 |
| Manufacturing | 0.024 | 0.149 | 0.159 | 0.874 | 1.024 | 0.764 - 1.372 |
| Construction | -0.088 | 0.393 | -0.224 | 0.823 | 0.916 | 0.424 - 1.977 |
| Transport and storage | 0.826 | 0.169 | 4.885 | <0.001 | 2.284 | 1.640 - 3.180 |
| Other | 0.249 | 0.197 | 1.264 | 0.206 | 1.283 | 0.872 - 1.887 |
| Gender*Age | -0.005 | 0.002 | -2.843 | 0.005 | 0.995 | 0.991 - 0.998 |
| Sector*Age |  |  |  |  |  |  |
| Education*Age | 0.009 | 0.004 | 2.340 | 0.019 | 1.009 | 1.002 - 1.017 |
| Health care*Age | -0.006 | 0.003 | -2.035 | 0.042 | 0.994 | 0.989 - 1.000 |
| Government*Age | 0.010 | 0.003 | 3.195 | 0.001 | 1.010 | 1.004 - 1.016 |
| Accommodation and food service*Age | 0.006 | 0.006 | 1.138 | 0.255 | 1.006 | 0.995 - 1.017 |
| Distributive trade*Age | 0.001 | 0.003 | 0.201 | 0.841 | 1.001 | 0.995 - 1.006 |
| Manufacturing*Age | -0.002 | 0.003 | -0.574 | 0.566 | 0.998 | 0.993 - 1.004 |
| Construction*Age | 0.005 | 0.003 | 1.719 | 0.086 | 1.005 | 0.999 - 1.012 |
| Transport and storage*Age | -0.003 | 0.003 | -1.079 | 0.280 | 0.997 | 0.991 - 1.003 |
| Other*Age | -0.006 | 0.004 | -1.623 | 0.105 | 0.994 | 0.987 - 1.001 |
| Gender*Sector |  |  |  |  |  |  |
| Gender* Education | -0.439 | 0.115 | -3.809 | <0.001 | 0.644 | 0.514 - 0.808 |
| Gender* Health care | -0.160 | 0.077 | -2.084 | 0.037 | 0.852 | 0.733 - 0.991 |
| Gender* Government | -0.324 | 0.085 | -3.830 | <0.001 | 0.723 | 0.613 - 0.854 |
| Gender* Accommodation and food service | -0.085 | 0.143 | -0.589 | 0.556 | 0.919 | 0.694 - 1.217 |
| Gender* Distributive trade | -0.334 | 0.085 | -3.950 | <0.001 | 0.716 | 0.607 - 0.845 |
| Gender* Manufacturing | -0.212 | 0.093 | -2.276 | 0.023 | 0.809 | 0.674 - 0.971 |
| Gender* Construction | 0.099 | 0.371 | 0.266 | 0.790 | 1.104 | 0.533 - 2.286 |
| Gender* Transport and storage | -0.352 | 0.113 | -3.123 | 0.002 | 0.703 | 0.564 - 0.877 |
| Gender* Other | -0.090 | 0.115 | -0.783 | 0.434 | 0.914 | 0.730 - 1.145 |
| Constant | -4.142 | 0.126 | -32.830 | <0.001 | 0.016 | 0.012 - 0.020 |

SE= standard error

CI = confidence interval
